# The Lure of Beauty: People Select Representations of Statistical Information Largely Based on Attractiveness, Not Comprehensibility

**DOI:** 10.1177/0272989X231201579

**Published:** 2023-10-23

**Authors:** Wolfgang Gaissmaier, Kevin E. Tiede, Rocio Garcia-Retamero

**Affiliations:** Department of Psychology, University of Konstanz, Konstanz, Germany; Centre for the Advanced Study of Collective Behaviour, University of Konstanz, Germany; Department of Psychology, University of Konstanz, Konstanz, Germany; Center for Adaptive Rationality, Max Planck Institute for Human Development, Berlin, Germany; Graduate School of Decision Sciences, University of Konstanz, Germany; Department of Experimental Psychology, University of Granada, Granada, Spain

**Keywords:** risk communication, icon arrays, medical decision making, graph literacy, numeracy

## Abstract

**Objective:**

People differ in whether they understand graphical or numerical representations of statistical information better. However, assessing these skills is often not feasible when deciding which representation to select or use. This study investigates whether people choose the representation they understand better, whether this choice can improve risk comprehension, and whether results are influenced by participants’ skills (graph literacy and numeracy).

**Methods:**

In an experiment, 160 participants received information about the benefits and side effects of painkillers using either a numerical or a graphical representation. In the “no choice” condition, the representation was randomly assigned to each participant. In the “choice” condition, participants could select the representation they would like to receive. The study assessed gist and verbatim knowledge (immediate comprehension and recall), accessibility of the information, attractiveness of the representation, as well as graph literacy and numeracy.

**Results:**

In the “choice” condition, most (62.5%) chose the graphical format, yet there was no difference in graph literacy or numeracy (nor age or gender) between people who chose the graphical or the numerical format. Whereas choice slightly increased verbatim knowledge, it did not improve gist or overall knowledge compared with random assignment. However, participants who chose a representation rated the representation as more attractive, and those who chose graphs rated them as more accessible than those without a choice.

**Limitations:**

The sample consisted of highly educated undergraduate students with higher graph literacy than the general population. The task was inconsequential for participants in terms of their health.

**Conclusions:**

When people can choose between representations, they fail to identify what they comprehend better but largely base that choice on how attractive the representation is for them.

**Highlights:**

- People differ systematically in whether they understand graphical or numerical representations of statistical information better. However, assessing these underlying skills to get the right representation to the right people is not feasible in practice. A simple and efficient method to achieve this could be to let people choose among representations themselves.
- However, our study showed that allowing participants to choose a representation (numerical v. graphical) did not improve overall or gist knowledge compared with determining the representation randomly, even though it did slightly improve verbatim knowledge.
- Rather, participants largely chose the representation they found more attractive. Most preferred the graphical representation, including those with low graph literacy.
- It would therefore be important to develop graphical representations that are not only attractive but also comprehensible even for people with low graph literacy.

Making informed health decisions requires understanding of basic statistical information about the benefits and side effects of medical treatments and other health-relevant behaviors. It is well documented, however, that understanding statistical information is quite challenging for most people, including for experts.^1–5^ It is therefore an important area of research how statistical information can be communicated more effectively. A promising avenue in this regard are graphical representations of statistical information, which can improve understanding of medical risks and promote healthy behaviors.^6–9^

However, while the use of graphical representations is often recommended as an aid for the interpretation of numerical data,^9,10^ one important caveat is that graphical representations cannot be assumed to be intuitively understandable by everyone. Rather, people differ in how well they understand graphical representations. This competency has been coined as *graph literacy*^11–13^ and is distinct from the ability to understand numerical information (*numeracy*^3,5^). In fact, research showed that people with low graph literacy may actually be better off with mere numbers.^14^

The question thus arises how to get the right representation to the right person, that is, a representation (numerical or graphical) that this person understands well. Assessing patients’ graph literacy and numeracy prior to presenting medical information is time-consuming and potentially awkward, so that it is often not feasible in practice. A much simpler and more feasible method consists in letting people choose which representation they would like to receive themselves, rather than deciding it for them. To improve knowledge, this method would require that people are able to identify the representation that they comprehend better, that is, the representation that fits to their graph literacy and numeracy skills. In this study, we therefore investigate whether allowing people to choose a representation of statistical information does indeed improve knowledge and increases the fit to the underlying skills, compared with a situation in which the representation is randomly assigned.

To investigate this question, we conducted an experiment in which participants received statistical information about the benefits and side effects of a painkiller medication. The central manipulation was that half of the participants could choose whether they wanted a graphical or a numerical representation of that information, whereas the other half of the participants received 1 of the 2 representations at random. At 2 time points of measurement, we assessed how well they comprehended (T1) and recalled (T2) the statistical information that they received. The 2 time points were primarily used to enhance the sensitivity to detect differences by avoiding potential ceiling effects and to check potential effects for robustness. In line with previous research (e.g., Gaissmaier et al.,^14^ Hawley et al.,^15^ Tait et al.,^16^ and Tiede et al.^17^), we assessed 2 different types of knowledge: verbatim knowledge (i.e., comprehension and recall of the precise knowledge about the risks and benefits of treatments) and gist knowledge (i.e., comprehension and recall of the essential information^18^). Further, we assessed participants’ ratings of subjective accessibility of the information and the attractiveness of its representation.

Three research questions guided us, which will be described in detail subsequently: 1) Which representation will be chosen predominantly, and who chooses the graphical rather than the numerical representation? 2) Does being able to choose between numerical and graphical representations foster knowledge of statistical information in comparison with random allocation to either graphical or numerical representations? 3) Does choice increase ratings of accessibility and/or attractiveness, suggesting that either of those attributes may play a role in choosing?

## What Will Be Chosen Predominantly, and Who Chooses Graphs Rather than Numbers?

Based on the existing literature, we can expect that most people will choose the graphical rather than the numerical representation: graphs are generally preferred,^19,20^ and they are rated as more attractive, helpful, or likeable in comparison with numbers,^14,16,21–23^ which even holds true for people with low graph literacy.^14^

But do people—despite this general preference for graphical representations—choose the representation that fits to their skills such as graph literacy and numeracy, at least to some degree? If this were the case, then people who select graphical rather than numerical representations should have higher graph literacy on average, because research has shown that people with high graph literacy benefit particularly from graphical representations.^7,13,14,24,25^ People choosing graphical representations may additionally have lower numeracy on average, because it is particularly those with low numeracy who benefit from graphical representations.^26,27^

Conversely, people higher in numeracy prefer to receive numerical instead of verbal information^28,29^ and may thus also prefer numbers over graphs. Furthermore, it has been shown that people with low numeracy focused on graphical information much earlier than people with high numeracy,^30^ which makes it plausible to assume that people with low numeracy would also be more likely to choose graphical representations. Finally, people seem to have some insight into their own graph literacy and numeracy skills, given that people’s subjectively rated graph literacy and numeracy correlate well with the actual abilities.^11,19,28,29^

## Does Being Able to Choose a Representation Foster Knowledge of Statistical Information?

If people who can choose between representations actually succeeded in choosing the representation that they understand better, they should perform better when confronted with knowledge questions compared with people who randomly received either of the representations, and this should hold true for both individuals who choose graphical and individuals who choose numerical representations. Previous research has studied whether people better understand formats they preferred and found mixed results. While some studies found that preferences were unrelated to knowledge,^31–34^ others have found that people fare better with formats they preferred.^35,36^

However, all of these conclusions were based on correlational results. In addition, in most studies, participants provided their preference ratings for all representations, these ratings did not have any consequences, and they were provided only after working with multiple different formats. However, letting people work with all representations before choosing would not be a feasible solution for everyday medical decision making. In sum, to the best of our knowledge, there is no study that has directly tested whether providing the opportunity to choose a representation prior to a knowledge task poses a promising approach to improve knowledge of medical information.

## Does Choice Increase Ratings of Accessibility and/or Attractiveness of the Representation?

Subjective ratings of the accessibility of the information as well as of the attractiveness of the representation could yield additional insights into how representations are chosen. As mentioned above, graphical representations have generally been found to be rated as more attractive than numerical ones (e.g., Gaissmaier et al.^14^). If choice were based on attractiveness, attractiveness ratings should generally be higher when people choose their representation, for both numbers and graphs. If choice were based on what people believe to understand well, then this could be reflected not only in higher actual performance but also in higher perceived accessibility of the information.

## Methods

### Design and Procedure

**Figure 1:**
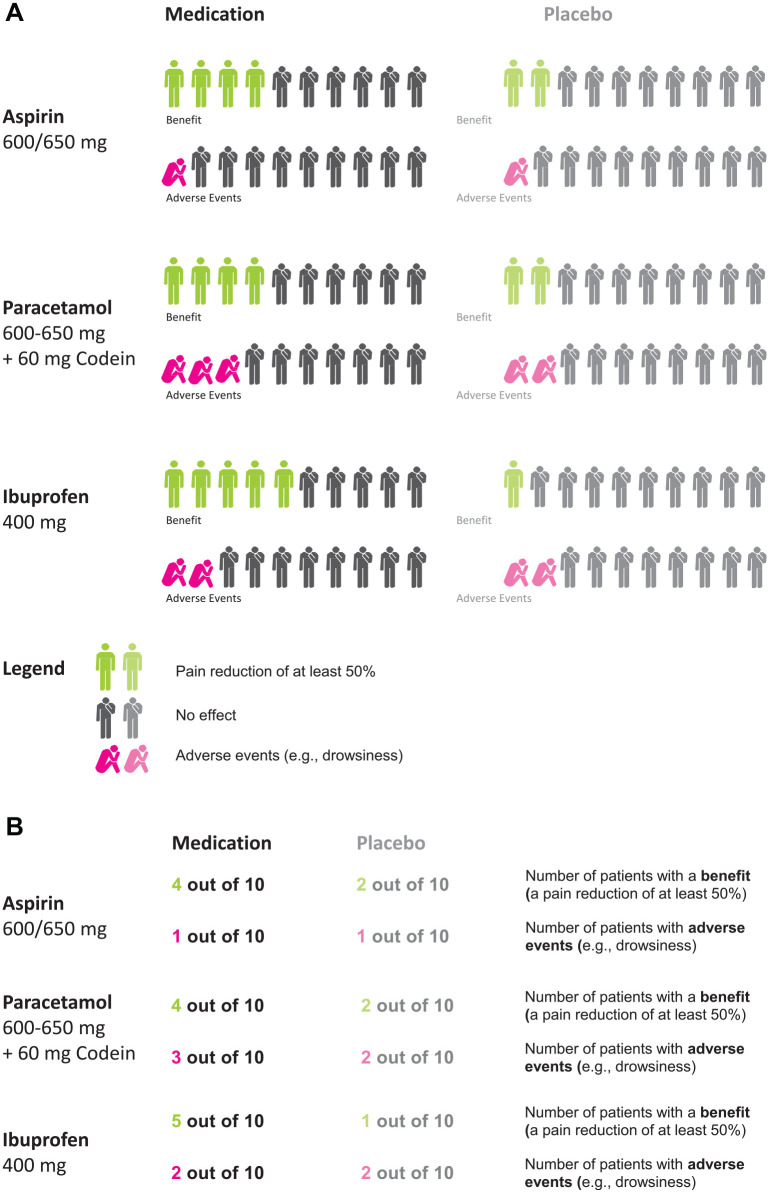
Illustration of the stimuli: the graphical (panel A) and numerical (panel B) representation of the statistical information about the painkillers.

The study was approved by the ethics committee of the Max Planck Institute for Human Development and the University of Granada. Participants were provided with information about the frequency of benefits and side effects of 3 painkilling medications (i.e., aspirin, ibuprofen, and paracetamol) in comparison with a placebo. The clinical evidence was taken from 3 Cochrane reviews,^37–39^ and this medical information has been previously used by Gaissmaier et al.^14^ and Tiede et al.^17^ The language of the study was Spanish, and all questions were asked with a pen-and-paper questionnaire. The information participants received on the painkillers was either represented numerically or graphically (see Figure 1).

After providing informed consent and stating demographic characteristics, participants were randomly assigned to the “no choice” or “choice” conditions and were provided with information about the medications. Within the “no choice” condition, half of the participants were randomly assigned to receive the numerical representation of the information, while the other half was randomly assigned to receive the graphical representation of the information. Within the “choice” condition, participants saw a brief example of what the numerical and graphical representation would look like and could decide which one they wanted to receive.

All participants received an actual printout of the information, either as numerical or graphical representation, and were asked a series of questions about the medication with the printout directly in front of them (T1). These questions concerned comprehension of the information, subjective evaluations of perceived accessibility of the information, and subjective attractiveness ratings of the representation (see the “Measures” section for details on those and all subsequent variables). Next, participants returned the printout with the representation to the experimenter and filled out the graph literacy and numeracy scale. Then, there was a 120 min break. During this break, participants attended to a lecture and a practice exercise about a topic unrelated to the current research (healthy nutrition in adolescents). After this break, participants received a task in which they had to recall the same knowledge questions that they had answered previously but without being provided with the information (T2).

In sum, the independent variables were condition (“no choice” v. “choice”), whether participants worked with the numerical or the graphical representation of the information, and their graph literacy. The dependent variables were comprehension and recall of both verbatim and gist knowledge (and we generally use the term *knowledge* if we average across comprehension [T1] and recall [T2]), (perceived) accessibility of the information, and attractiveness of the representation; numeracy was included as a covariate.

### Measures

All knowledge questions as well as subjective ratings were previously used by Gaissmaier et al.^14^ and Tiede et al.^17^ For the knowledge, graph literacy, and numeracy questionnaires, missing answers were treated as incorrect. We tested whether this scoring of missing answers affected the results compared with scoring them as missing data. Overall, there were only few missing answers, and the results did not depend on scoring scheme (for details, see Supplementary Materials).

#### Gist knowledge

Gist knowledge reflects the essential understanding of the information and was assessed with 5 qualitative questions that were nonnumerical and asked for ordinal comparisons between the painkillers (e.g., “Which drug caused side effects least frequently?”“Which painkiller was worst overall?”). To answer these questions correctly, participants needed to consider the differences a medication made in comparison with a placebo group; for instance, whether side effects were more frequently observed in the medication compared with the placebo group. This placebo scoring is therefore how we scored the answers to the gist knowledge question for the main text. However, participants may have erroneously looked at only the medication condition, ignoring the placebo condition. Therefore, we also checked the results with an alternative medication-only scoring scheme and will report only its most central findings in the main text, whereas the details are reported in the Supplementary Materials. With both scoring methods, the gist knowledge score is the average proportion of correct answers and was assessed with identical questions at T1 and T2. Missing cells were treated as mistakes.

#### Verbatim knowledge

Verbatim knowledge reflects the precise quantitative understanding and was assessed with 8 quantitative questions that asked for numerical statements and comparisons. To answer those questions, participants needed to read off frequencies from the information chart (e.g., “How many patients experience side effects with Ibuprofen?”) and compute absolute differences between 2 frequencies from the information chart (e.g., “How many patients experience a benefit of ibuprofen that they would not have had with a placebo?”). The verbatim knowledge score is the average proportion of correct answers and was assessed with identical questions at T1 and T2. Missing cells were treated as mistakes.

#### Accessibility

Subjective accessibility of the information was assessed with 5 questions, each of which could be answered on a 5-point scale ranging from 1 (*not at all*) to 5 (*very much*). They covered the following aspects: comprehensibility, usefulness, seriousness, intuitive accessibility, and difficulty of answering the questions. Answers were averaged to generate 1 accessibility score for each participant, excluding missing items. The internal consistency of this scale was acceptable (Cronbach’s α = 0.66).

#### Attractiveness

Subjective attractiveness of the representation was assessed with 8 questions, each of which could be answered on a 5-point scale ranging from 1 (*not at all attractive*) to 5 (*very attractive*). They covered the following aspects of the representation: overall impression, attractiveness of colors, imagery, technical implementation, size, font size, font, and composition. Answers were averaged to receive 1 attractiveness score for each participant, excluding missing items. The internal consistency of this scale was good (Cronbach’s α = 0.87).

#### Graph literacy

We assessed graph literacy using the Spanish translation of the Graph Literacy Scale. The scale assesses an individual’s knowledge of health-related information based on graphical representations with 13 items on 3 levels of difficulty: reading the data (i.e., finding the specific information on a graph; 4 items), reading between the data (i.e., understanding relationships in the data shown in the graph; 4 items), and reading beyond the data (i.e., making inferences and predictions from the presented data; 5 items). The English and German versions of this scale were validated on nationally representative samples in Germany and the United States and showed good psychometric properties.^12^ The Spanish version was translated carefully using a back-translation method^40^ and has been successfully used in other studies (e.g., Okan et al.^13^). The graph literacy score corresponds to the proportion of questions answered correctly.

#### Numeracy

We assessed numeracy as a control variable, because it is correlated with graph literacy (in our sample: *r*[154] = 0.25, *P* = 0.002).^12^ Numeracy was assessed using a 9-item questionnaire that consisted of 3 items by Schwartz et al. and 6 items by Lipkus et al.^41^ The scale assesses an individual’s ability to use and process probabilistic and numerical concepts. For example, one item was, “If the chance of getting a disease is 10%, how many people would be expected to get the disease out of 1,000?” This scale has been validated on nationally representative samples.^11^ The numeracy score corresponds to the proportion of questions answered correctly.

### Participants

One hundred sixty participants (80 in each of the conditions “choice” and “no choice”) were included in the study that comprised working with the materials (T1) and the recall test (T2). All participants were students at the University of Granada, Spain, and they received course credit for participation. The sample included 78 females and was *M* = 24.0 y old (*s* = 4.1 y). Mean graph literacy was 0.81 (*s* = 0.11) and mean numeracy was 0.83 (*s* = 0.18).

## Results

The data and analysis script for this study are openly available at https://osf.io/c94hj/?view_only=38e293b9ff9c47a7af624361898cda7a.

### Participant Characteristics

As presented in Table 1, participant characteristics did not differ between the conditions “no choice” and “choice.”

**Table 1:** Participant Characteristics and Descriptive Statistics for Conditions “No Choice” and “Choice”

	No Choice	Choice
Participant characteristics		
*N*	80	80
Mean age, y (*s*)	24.10 (4.29)	23.94 (3.97)
% Female	51^a^	48
Mean graph literacy (*s*)	0.83 (0.09)	0.83 (0.11)
Mean numeracy (*s*)	0.85 (0.17)	0.82 (0.18)
Descriptive statistics		
Mean gist knowledge T1 (*s*)	0.86 (0.15)	0.83 (0.15)
Mean gist knowledge T2 (*s*)	0.74 (0.26)	0.70 (0.26)
Mean verbatim knowledge T1 (*s*)	0.76 (0.24)	0.84 (0.19)
Mean verbatim knowledge T2 (*s*)	0.48 (0.17)	0.50 (0.20)
Mean accessibility (*s*)	3.22 (0.65)	3.43 (0.71)
Mean attractiveness (*s*)	3.35 (0.74)	3.70 (0.80)

a This percentage is based on *n* = 78, as 2 participants did not indicate gender.

### What Is Chosen Predominantly, and Who Chose Graphs Rather than Numbers?

We first checked whether participants in the “choice” condition were more likely to choose one of the representations by comparing the actual choice proportions with a 50/50 split, using a binomial test. Participants were generally more likely to choose the graphical representation than the numerical one. Of the 80 participants in this condition, there were 50 participants (62.5%) who chose the graphical representation but only 30 (37.5%) who chose the numerical representation, which we compared to 50% each expected by chance with a binomial test, *P* = 0.033.

Within the “choice” condition, we then tested whether participants who chose the graphical condition had higher graph literacy and lower numeracy scores than those who chose the numerical representation, using *t* tests. As presented in Table 2, this was not the case: in the “choice” condition, the participants who chose the numerical representation did not differ from the participants who chose the graphical representation. This held true for graph literacy, *t*_Welch_(43.49) = 0.820, *P* = 0.42, numeracy, *t*(76) = 0.420, *P* = 0.68, age, *t*(78) = 0.528, *P* = 0.60, and gender, χ^2^(1, *n* = 80) = 0.013, *P* = 0.91, respectively.

**Table 2:** Participant Characteristics and Descriptive Statistics of Participants Who Chose the Numerical and Graphical Representation in the “Choice” Condition (*n* = 80)

	Numerical	Graphical
Participant characteristics		
*N*	30	50
Mean age, y (*s*)	23.63 (4.37)	24.12 (3.75)
% Female	50	46
Mean graph literacy (*s*)	0.83 (0.13)	0.82 (0.09)
Mean numeracy (*s*)	0.83 (0.19)	0.81 (0.18)
Descriptive statistics		
Mean gist knowledge T1 (*s*)	0.86 (0.09)	0.81 (0.17)
Mean gist knowledge T2 (*s*)	0.76 (0.24)	0.64 (0.26)
Mean verbatim knowledge T1 (*s*)	0.88 (0.19)	0.83 (0.19)
Mean verbatim knowledge T2 (*s*)	0.54 (0.21)	0.47 (0.19)
Mean accessibility (*s*)	3.20 (0.71)	3.57 (0.68)
Mean attractiveness (*s*)	3.43 (0.77)	3.86 (0.77)

### Does Choice Foster Knowledge?

To analyze whether knowledge differed between the “no choice” and the “choice” condition, we ran a linear mixed-effects model with random intercepts for participants. The outcome variable was knowledge and the predictors were time (T1 v. T2; effect-coded as T1 = −0.5, T2 = +0.5) and knowledge type (verbatim v. gist; effect coded as verbatim = −0.5, gist = +0.5), the between-subjects factors condition (“no choice” v. “choice”; effect coded as no choice = −0.5, choice = +0.5), representation (numbers v. graphs; effect coded as numbers = −0.5, graphs = +0.5), graph literacy (centered), and all possible interactions. Numeracy (centered) was included as a covariate. Note that including graph literacy as a predictor variable, yet numeracy only as a covariate, was done in line with a related study,^14^ based on which we expected an important interaction of graph literacy with representation, whereas for numeracy, we merely expected a general effect on knowledge, but no interactions. Controlling for numeracy is still important, because it is correlated with graph literacy, and this procedure allowed us to assess effects of graph literacy that go above and beyond numeracy.

As a robustness check, we nevertheless also ran the same model with numeracy as a predictor (see Supplementary Materials, Table S4). In that model, the results were similar if not explicitly mentioned. Four participants did not fill out the numeracy questionnaire, and due to row-wise deletion of missing data in the mixed-effects models, they were not included in the analysis that included numeracy as a predictor or covariate. We also tested whether our results were robust to including these 4 participants but excluding numeracy as a covariate and found results similar to those reported below.

**Figure 2:**
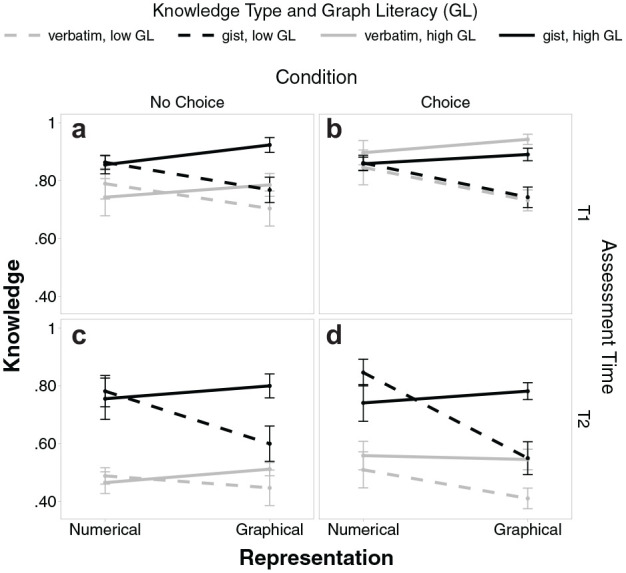
Gist and verbatim knowledge scores for numbers versus graphs with separate lines for people with high and low graph literacy, separate panels for T1 (upper panels a and b) and T2 (lower panels c and d), and separate panels for conditions “no choice” (left panels a and c) and “choice” (right panels b and d). Error bars represent 1 standard error of the mean.

Furthermore, for a better interpretation of the interaction of 2 predictors, we additionally tested simple effects by analyzing the effect of 1 predictor separately in the subgroups of the other predictor. We use a mixed-effects model because it enables us to both account for within-subjects factors (i.e., time and knowledge type) and to include a continuous predictor (i.e., graph literacy), as suggested by Magezi.^42^ Figure 2 illustrates the results, and Table A1 in the Appendix presents the full results.

#### Effects of graph literacy and numeracy

People higher in graph literacy had overall higher knowledge (high: $\bar{x}$ = 73.4, *s* = 13.4 v. low: $\bar{x}$ = 70.3, *s* = 14.6; *b*_GraphLiteracy_ = 0.35, *SE* = 0.10, *P* < 0.001). People higher in numeracy, however, had slightly lower overall knowledge (high: $\bar{x}$ = 70.3, *s* = 15.2 v. low: $\bar{x}$ = 72.1, *s* = 13.7, *b*_Numeracy_ = −0.13, *SE* = 0.06, *P* = 0.036).

#### Effects of choice

In contrast to our expectations, choice did not generally improve knowledge (choice: $\bar{x}$ = 71.7, *s* = 13.3 v. no choice: $\bar{x}$ = 70.8, *s* = 15.2; *b*_Condition_ = 2.74, *SE* = 2.16, *P* = 0.21). Importantly, simple effects analyses revealed that choice improved knowledge neither for people who saw graphs (choice: $\bar{x}$ = 68.9, *s* = 14.2 v. no choice: *$\bar{x}$* = 69.6, *s* = 16.3; *b*_Condition_ = 0.64, *SE* = 2.98, *P* = 0.83) nor for people who saw numbers (choice: $\bar{x}$ = 76.5, *s* = 10.1 v. no choice: $\bar{x}$ = 71.9, *s* = 14.4; *b*_Condition_ = 4.89, *SE* = 3.09, *P* = 0.112; interaction: *b*_Condition×Representation_ = −4.40, *SE* = 4.31, *P* = 0.31).

Choice did, however, specifically increase verbatim knowledge (choice: $\bar{x}$ = 67.0, *s* = 16.6 v. no choice: $\bar{x}$ = 61.9, *s* = 17.6; *b*_condition_ = 6.76, *SE* = 2.72, *P* = 0.014), which was not the case for gist knowledge (choice: $\bar{x}$ = 76.5, *s* = 17.2 v. no choice: $\bar{x}$ = 79.6, *s* = 18.3; *b*_condition_ = −1.28, *SE* = 2.71, *P* = 0.64; interaction: *b*_Condition×KnowledgeType_ = −7.91, *SE* = 2.84, *P* = 0.006). Note, however, that the interaction disappeared in the model with numeracy as predictor rather than a covariate (*b*_Condition×KnowledgeType_ = −4.42, *SE* = 2.96, *P* = 0.136; see Table S4 in Supplementary Materials); thus, it should be interpreted with caution.

Interestingly, in contrast to (our default) placebo scoring, if we scored gist knowledge with medication-only scoring, choice increased overall knowledge (choice: $\bar{x}$ = 77.0, *s* = 13.3 v. no choice: $\bar{x}$ = 73.9, *s* = 14.6; *b*_Condition_ = 4.73, *SE* = 2.08, *P* = 0.023), but there was no interaction between condition and knowledge type (*b*_Condition×KnowledgeType_ = −4.00, *SE* = 2.84, *P* = 0.159). Simple effects analyses revealed that this overall effect of choice was again exclusively driven by the same specific effect of choice on verbatim knowledge (choice: $\bar{x}$ = 67.0, *s* = 16.6 v. no choice: $\bar{x}$ = 61.9, *s* = 17.6; *b*_condition_ = 6.76, *SE* = 2.72, *P* = 0.014), in the absence of an effect on gist knowledge (choice: = 87.0, *s* = 16.8 v. no choice: $\bar{x}$ = 86.0, *s* = 16.6; *b*_condition_ = 2.69, *SE* = 2.42, *P* = 0.27). In the end, we thus consider the results of medication-only scoring to be similar to placebo scoring.

#### Effects of representation (numerical, graphical) and its interaction with graph literacy

Participants who worked with numbers were overall better than those who worked with graphs (numbers: $\bar{x}$ = 73.9, *s* = 12.9 v. graphs: $\bar{x}$ = 69.2, *s* = 15.0, *b*_Representation_ = −4.88, *SE* = 2.15, *P* = 0.025). As illustrated in Figure 2 and replicating Gaissmaier et al.,^14^ the effect of representation was different for people with low versus high graph literacy (interaction: *b*_GraphLiteracy×Representation_ = 0.74, *SE* = 0.20, *P* < 0.001). Simple effects analyses revealed that specifically people with low graph literacy performed better with numbers than with graphs (numbers: $\bar{x}$ = 76.2, *s* = 10.3 v. graphs: $\bar{x}$ = 66.7, *s* = 15.7; *b*_Representation_ = −12.21, *SE* = 3.20, *P* < 0.001), whereas those with high graph literacy did not reliably perform better with graphs than with numbers (numbers: $\bar{x}$ = 70.3, *s* = 15.6 v. graphs: $\bar{x}$ = 77.4, *s* = 8.6; *b*_Representation_ = 3.72, *SE* = 2.74, *P* = 0.179).

### Does Choice Increase Ratings of Accessibility and/or Attractiveness?

**Figure 3:**
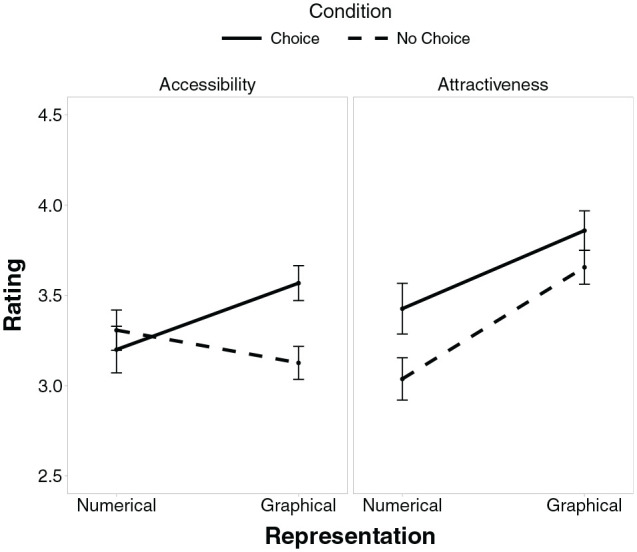
Accessibility (a) and attractiveness (b) scores for numbers versus graphs with separate lines for conditions “no choice” and “choice.” Error bars represent 1 standard error of the mean.

To analyze whether accessibility and attractiveness differed between the “no choice” and “choice” condition, we ran 2 separate linear regression models with accessibility and attractiveness as outcome variables, respectively; the predictors were condition (“no choice” v. “choice”; effect coded), representation (numbers v. graphs; effect coded), and graph literacy (centered) and their interactions. Numeracy (centered) was included as a covariate but did not yield any effect on accessibility and attractiveness (with betas being basically zero) and is thus not further discussed in detail here. For both models, the results were similar when running a model with numeracy as predictor and when excluding numeracy as a covariate or predictor. The results are illustrated in Figure 3.

Participants’ ratings of accessibility did not differ between graphs and numbers (numbers: $\bar{x}$ = 3.26, *s* = 0.70 v. graphs: $\bar{x}$ = 3.37, *s* = 0.67; *b*_Representation_ = 0.08, *SE* = 0.11, *P* = 0.46) and did not depend on graph literacy (high: $\bar{x}$ = 3.29, *s* = 0.69 v. low: $\bar{x}$ = 3.36, *s* = 0.69, *b*_GraphLiteracy_ = 0.00, *SE* = 0.01, *P* = 0.70). Choice did not generally result in enhanced accessibility (choice: $\bar{x}$ = 3.43, *s* = 0.71 v. no choice: $\bar{x}$ = 3.22, *s* = 0.65; *b*_Condition_ = 0.17, *SE* = 0.11, *P* = 0.125). Choice did, however, lead to enhanced accessibility of the graphical representation in comparison to no choice (choice: $\bar{x}$ = 3.57, *s* = 0.68 v. no choice: $\bar{x}$ = 3.13, *s* = 0.58; *b*_Condition_ = 0.46, *SE* = 0.14, *P* = 0.002), which was not the case for the numerical representation (choice: $\bar{x}$ = 3.20, *s* = 0.71 v. no choice: $\bar{x}$ = 3.31, *s* = 0.71; *b*_Condition_ = −0.11, *SE* = 0.18, *P* = 0.52; interaction: *b*_Condition×Representation_ = 0.57, *SE* = 0.22, *P* = 0.012).

Participants generally rated graphs as more attractive than numbers (numbers: $\bar{x}$ = 3.20, *s* = 0.77 v. graphs: $\bar{x}$ = 3.77, *s* = 0.70; *b*_Representation_ = 0.52, *SE* = 0.12, *P* < 0.001), but attractiveness ratings did not depend on graph literacy (high: $\bar{x}$ = 3.50, *s* = 0.74 v. low: $\bar{x}$ = 3.55, *s* = 0.83; *b*_GraphLiteracy_ = 0.00, *SE* = 0.01, *P* = 0.48). Choice generally resulted in enhanced attractiveness, (choice: $\bar{x}$ = 3.70, *s* = 0.80 v. no choice: $\bar{x}$ = 3.35, *s* = 0.74; *b*_Condition_ = 0.30, *SE* = 0.12, *P* = 0.015). This effect did not seem to differ between the numerical and graphical representation (*b*_Condition×Representation_ = −0.19, *SE* = 0.24, *P* = 0.43). However, when analyzing the effect of choice separately for numerical and graphical representation, the effect of choice was significant only for the numerical representation (choice: $\bar{x}$ = 3.43, *s* = 0.77 v. no choice: $\bar{x}$ = 3.04, *s* = 0.74; *b*_Condition_ = 0.40, *SE* = 0.19, *P* = .040) but not for the graphical representation (choice: $\bar{x}$ = 3.86, *s* = 0.77 v. no choice: $\bar{x}$ = 3.66, *s* = 0.59; *b*_Condition_ = 0.21, *SE* = 0.15, *P* = 0.188).

## Discussion

Not everyone benefits from graphical representations of statistical information, and for some people, numerical representations are actually better suited to foster knowledge.^14,24^ Graph literacy differentiates those who benefit from graphical representations from those who do not. However, in practice, for instance, in a patient-physician consultation, it is not feasible to assess graph literacy to determine who should receive graphical representations; this assessment would be time-consuming and may be perceived as awkward (or even scary). Thus, the question emerged of how it could alternatively be decided who receives which kind of representation. A simple and potentially effective way would be to allow people to choose themselves which kind of representation they prefer.

We were first asking which representation gets chosen more often and whether people chose representations in line with underlying skills, particularly graph literacy, but also numeracy. In line with previous research, most participants chose the graphical over the numerical representation (e.g., Nayak et al.^33^). However, this choice was clearly not based on individual skills such as graph literacy or numeracy: participants who chose the graphical representation did not differ with regard to graph literacy or numeracy from those who chose the numerical representation, and they also did not differ in terms of age and gender. Thus, even though it has been documented that people have a sense for their skills (by moderate correlations between objective and subjective measures of graph literacy and numeracy^4,19^), that does not seem to determine their choice for a representation. This finding is in line with a study that asked participants who worked with different representations to rank order their preferences for a numerical and 2 graphical representations and found no differences in graph literacy for rank-ordered preferences.^33^

Our second question was whether allowing people to choose the representation would yield better objective comprehension and recall of the information, which could be the case if choice fosters the match between people’s abilities and chosen representations. Our results, however, show that choosing does not increase comprehension and recall overall. To test whether this null effect could be attributed to limited power to detect a significant effect, we ran a simulation-based sensitivity analysis following DeBruine and Barr^43^ and found that given our sample and our data, we were able to detect a difference of at least 5.9 (6.8) percentage points in the knowledge score with 80% (90%) power. Thus, our study had been able to detect a substantial effect of choice on overall knowledge but not effects smaller than that. It is debatable whether effects smaller than about 6 percentage points would be clinically relevant in the first place, given baseline knowledge scores of about 70% (across types of knowledge and measurement times).

With regard to different types of knowledge, choice did not affect gist knowledge and recall at all, yet it slightly increased verbatim knowledge compared with the “no choice” condition. However, although this increase in verbatim knowledge was statistically significant, it is unclear whether this increase of about 5 percentage points on 1 knowledge dimension is clinically relevant given a baseline performance of about 70% (across both points in time). Furthermore, whereas having precise verbatim knowledge about clinical options is clearly important, gist knowledge may in fact matter more for making medical decisions.^15,44^ Finally, the effect of choice on verbatim knowledge was not perfectly robust to the exact specifications of the model, as it largely disappeared when including numeracy as predictor rather than as a covariate (see Table S4 in the Supplementary Materials). On the other hand, with medication-only scoring of gist knowledge, there was a reliable effect of choice on overall knowledge, even though the difference was only 3.1 percentage points. As this effect was again solely driven by the specific effect of choice on verbatim knowledge, similar to (our default) placebo scoring, this does not change our overall conclusion: there is a specific and small effect of choice on verbatim knowledge and recall. Yet more substantial, and more general, knowledge improvements would have been possible had participants based their choice more strongly on their skills, as predictable differences in performance we found illustrate: replicating the findings of Gaissmaier et al.^14^ and Garcia-Retamero and Galesic,^24^ performance with the different representations (numbers versus graphs) depended on graph literacy, such that particularly participants with low graph literacy achieved better gist and verbatim knowledge with the numerical than the graphical representation.

The third and final question was whether subjective ratings of accessibility and/or attractiveness would be increased in the “choice” condition, revealing potential motifs of choosing a particular representation. In line with the general preference for the graphical representation, attractiveness ratings of the graphical representation were higher than those of the numerical representation. Importantly, choice consistently increased ratings of attractiveness in comparison with the randomly allocated representations, for both numbers and graphs. This suggests that perceptions of how attractive particular representations are for individual participants played an important role when they chose between representations. Of course, there could additionally be an effect in the other direction, that is, from choice to attractiveness. It is very well documented that people judge options to be more desirable after they have chosen them, even if desirability was identical before making the choice.^45^ As the association between choice and attractiveness is merely a correlation in these data, we cannot be certain about the direction of the effect. However, it speaks against mere postdecision effects that accessibility ratings were not also generally increased, but future research would be needed to tell more precisely.

Whereas choice did not generally increase rated accessibility, it specifically increased ratings of accessibility for the graphical representation. Yet this increased accessibility did not translate into increased overall knowledge scores, as summarized above. In fact, when looking at bivariate correlations between ratings of accessibility and the various knowledge scores (gist and verbatim knowledge at T1 and T2, respectively), it turns out that they are, by and large, uncorrelated with one another; in one case, there is even one small negative correlation, indicating lower gist knowledge at T1 with higher ratings of accessibility (*r* = −0.19, *P* = 0.017).

To summarize, the choice between a graphical and a numerical representation 1) revealed a general preference for the graphical representation, and this preference was not related to abilities (graph literacy and numeracy, respectively); 2) did not yield better comprehension and recall overall; and 3) was correlated with increased attractiveness ratings for chosen representations compared with their randomly allocated counterparts and for graphs additionally with increased perceived accessibility (without translating into increased knowledge). Taken together, letting patients choose the representation is not an efficient way to ensure they receive a representation that they understand or recall well. Although choice improved verbatim knowledge, this limited benefit is not enough to recommend giving patients the opportunity to choose a representation as an approach to improve knowledge of medical information. Rather, these results suggest that people choose the representation they thought of as more attractive and, at least for graphs, the one they believe to more accessible, rather than choosing the one that they objectively comprehend better.

### Limitations

Several limitations of the study need to be taken into consideration when interpreting the results. First, the sample consisted of a diverse yet highly educated group of students who are not representative of the general population in Spain. Consequently, they have higher graph literacy scores than an average person randomly drawn from the population (0.81 compared with ≈0.72 in nationally representative samples in Germany and the United States^12^). On one hand, this prohibits generalization to the overall public. On the other hand, it is actually striking that even within such a limited range of graph literacy, substantial differences in understanding the graphical representation of the statistical information can be observed, and we could replicate the findings of Gaissmaier et al.,^46^ which had a more diverse sample. It seems plausible to assume that those differences would be larger in the general population, but this is, of course, an empirical question.

The second limitation is that the information presented to participants was not of personal relevance to them. That is, they could choose a representation independent of how well they understood it without having to fear any real consequences. The results on whether (typically financial) incentives make a difference for behavior are mixed,^46^ so that we cannot know whether the results would be different in an actual choice situation. This makes it all the more important to study the choices of people who face real medical decisions in the future.

Furthermore, there are some peculiarities in the data that we would like to discuss transparently. The first peculiarity is that numeracy was not at all related to comprehension, recall, or accessibility ratings (and in some cases even negatively so, even though with a tiny effect size). This is surprising in light of the vast literature showing the impact of numeracy on understanding health-related statistical information^1,9^ but also in light of its correlation with graph literacy in this data (*r* = 0.25), which is somewhat lower than what has been observed elsewhere^12,14^ but not zero and/or completely out of range. With a very high average proportion correct on the numeracy scale (0.83), a potential ceiling effect could be an explanation, even though this did not occur for graph literacy with a similarly high average proportion correct (0.81). However, numeracy was much more skewed than graph literacy, with the most frequently observed score being a perfect numeracy score (achieved by about a third of participants; see Figure S2 in the Supplementary Materials). This may, at least in part, explain why we did not find the expected effect of numeracy.

The second peculiarity is that comprehension and recall were, on average, better with the numerical than with the graphical representation. Whereas a substantial amount of literature suggests that graphical representations are helpful to understand statistical information,^7–9^ other research challenges the assumption that graphical representations would be helpful for everyone but stresses the importance of graph literacy as a prerequisite in this regard.^14,24^ Also here, it was particularly those with low graph literacy for whom numbers turned out to be substantially better than graphs. This replication of this very specific interaction between graph literacy and the kind of representation (numbers v. graphs) generally underpins the quality and reliability of the data at hand. Yet it does not rule out that the specific graphical representation we used was less understandable than others and that also the results could thus be different for other graphical representations. In any case, the results highlight the need for designing more easily understandable graphical representations, which we will further discuss in the Conclusion.

## Conclusion

People differ in which kind of representation of statistical information they understand best: Some people understand graphical representations better, others are better off with mere numbers. Yet assessing their abilities to understand graphical and numerical information is infeasible in practice. Giving patients the opportunity to decide which representation to receive could have been a very efficient way to provide those information representations to patients that fit their abilities and thus improve comprehension and recall. However, despite some very minor benefits for verbatim knowledge, overall this approach is not sufficient to improve comprehension and recall of medical information. Instead, people seem to choose the representation they perceive as more attractive. Thus, improving knowledge by getting the right representation to the right people would require other methods of tailoring rather than choice, yet those would need to be feasible in practice. As graphical representations were rated as being more attractive and are preferred by a majority, there is another solution to improve knowledge that requires further work, however: to develop better graphical representations that are so clearly designed that they are understandable for broader audiences—including for those with low graph literacy. Important steps in that direction include adding numerical and textual information to graphs that simply describes what can be seen, which has been shown to improve performance and reduce the impact of numeracy,^47,48^ as well as to improve knowledge of less graph-literate individuals.^49^

## Supplemental Material

sj-docx-1-mdm-10.1177_0272989X231201579 – Supplemental material for The Lure of Beauty: People Select Representations of Statistical Information Largely Based on Attractiveness, Not Comprehensibility Click here for additional data file. Supplemental material, sj-docx-1-mdm-10.1177_0272989X231201579 for The Lure of Beauty: People Select Representations of Statistical Information Largely Based on Attractiveness, Not Comprehensibility by Wolfgang Gaissmaier, Kevin E. Tiede and Rocio Garcia-Retamero in Medical Decision Making

## Appendix

**Table A1.** Results of the Linear Mixed-Effects Model with Random Intercepts for Participants

Predictor	*b*	*SE*	*P*
Intercept	72.10	1.08	**<0.001**
Condition (no choice v. choice)	2.74	2.16	0.21
Representation (numerical v. graphical)	−4.88	2.15	**0.024**
Graph literacy (GL)	0.35	0.10	**0.001**
Assessment time (time; knowledge v. recall)	−21.53	1.42	**<0.001**
Knowledge type (type; verbatim v. gist)	14.10	1.42	**<0.001**
Condition × representation	−4.40	4.31	0.31
Condition × GL	0.09	0.20	0.66
Condition × time	−3.35	2.84	0.24
Condition × type	−7.91	2.84	**0.006**
Representation × GL	0.74	0.20	**<0.001**
Representation × time	−3.11	2.84	0.28
Representation × type	−2.00	2.84	0.48
GL × time	−0.03	0.13	0.82
GL × type	0.07	0.13	0.60
Time × type	20.00	2.84	**<0.001**
Condition × representation × GL	0.13	0.40	0.76
Condition × representation × time	−2.64	5.68	0.64
Condition × representation × type	−0.05	5.68	0.99
Condition × GL × time	−0.07	0.26	0.79
Condition × GL × type	−0.36	0.26	0.177
Condition × time × type	8.15	5.68	0.152
Representation × GL × time	0.25	0.26	0.34
Representation × GL × type	0.34	0.26	0.200
Representation × time × type	−7.54	5.68	0.19
GL × time × type	0.24	0.26	0.36
Condition × representation × GL × time	−0.01	0.53	0.99
Condition × representation × GL × type	0.42	0.53	0.43
Condition × representation × time × type	1.72	11.37	0.88
Condition × GL × time × type	0.17	0.53	0.75
Representation × GL × time × type	0.66	0.53	0.21
5-way interaction	0.24	1.06	0.82
Numeracy	−0.13	0.06	**0.035**

*Note.* Results in boldface indicate significant predictors.
