# Supplementary material for: The Lure of Beauty: People Select Representations of Statistical Information Largely Based on Attractiveness, Not Comprehensibility
Source: Med Decis Making. 2023 Oct 23;43(7-8):774–88. doi: 10.1177/0272989X231201579 (PMC10625725; doi:10.1177/0272989X231201579)
Supplement: sj-docx-1-mdm-10.1177_0272989X231201579 – Supplemental material for The Lure of Beauty: People Select Representations of Statistical Information Largely Based on Attractiveness, Not Comprehensibility [file sj-docx-1-mdm-10.1177_0272989X231201579.docx]

**Supplemental Materials**

**to the Article**

**“The Lure of Beauty: People Select Representations of Statistical Information Largely Based on Attractiveness, not Comprehensibility”**

**Additional Analysis p. 2**

**Distribution of Graph Literacy and Numeracy p. 13**

**Materials Used in the Study p. 14**

**Additional Analysis**

In addition to the analyses presented in the main text, we also tested the robustness of our results. In the following, we present results based on alternative scoring schemes and models. First, recall that for answering gist knowledge questions correctly, participants needed to consider the difference between the medication and the placebo groups, respectively, which is why we used placebo scoring for the main analyses presented in the main text. As participants may have erroneously ignored the placebo condition and have only looked at the medication condition, we additionally present here the results of medication-only scoring of gist knowledge questions. Second, we tested whether scoring missing answers as missings (vs. incorrect) affects our results. Finally, we present result of a model variant which includes numeracy as a predictor (vs. covariate) in the mixed-effects models.

**Medication-only scoring of gist knowledge (as compared to placebo scoring, which is presented in the main text)**

In contrast to placebo scoring, medication-only scoring considers those answers to be correct that would be the right answer if one only looked at the medication condition and ignored the difference to the placebo condition. Even though this is a factually incorrect interpretation of the data, this may have been how some participants interpreted the data, because the concept of having to compare the medication to the placebo condition was unknown to them. To illustrate how the scoring methods can make a difference, consider the question which drug caused side effects least frequently and consult Figure 1. With placebo scoring, both Aspirin and Ibuprofen are considered correct, as there is no increase in side effects in the medication compared to the placebo group in both cases, even though the base rate of side effects is different (likely due to differences in study populations). With medication-only scoring, only Aspirin is considered correct. To test whether medication-only scoring alters the conclusions of our main analysis, we ran the main model on knowledge as outcome variable again, but with the new scoring of the gist-knowledge items.

The results are presented in Table S1 and Figure S1. Although the results are mostly similar to the results reported in the main text (see Table A1), they differ in terms of the effect of choice. Specifically, in contrast to placebo scoring, with medication-only scoring there now was an effect of condition (“no choice” vs. “choice”) on overall knowledge (choice: *M* = 77.0, *SD* = 13.3 vs. no choice: *M* = 73.9, *SD* = 14.6; *b*_Condition_ = 4.73, *SE* = 2.08, *p* = .023), but no interaction between condition and knowledge type (*b*_Condition×KnowledgeType_ = -4.00, *SE* = 2.84, *p* = .159). However, when examining the effect of choice on verbatim and gist knowledge separately, it becomes evident that the effect on overall knowledge is, again, exclusively driven by the effect of condition on verbatim knowledge, similar to placebo scoring. In particular, choice increased verbatim knowledge (because it was not affected by the change in scoring; as a reminder, choice: *M* = 67.0, *SD* = 16.6 vs. no choice: *M* = 61.9, *SD* = 17.6; *b*_condition_ = 6.76, *SE* = 2.72, *p* = .014), but not gist knowledge (choice: *M* = 87.0, *SD* = 16.8 vs. no choice: *M* = 86.0, *SD* = 16.6; *b*_condition_ = 2.69, *SE* = 2.42, *p* = .270). This pattern of simple effects is the same as for the results with placebo scoring reported in the main text.

Table S1. Results of the linear mixed-effects model with random intercepts for participants, based on data with the medication-only scoring scheme described above.

| Predictor | *b* | *SE* | *p* |
| --- | --- | --- | --- |
| Intercept | 76.19 | 1.03 | **< .001** |
| Condition (no choice vs. choice) | 4.73 | 2.08 | **.023** |
| Representation (numerical vs. graphical) | -4.81 | 2.07 | **.021** |
| Graph Literacy (GL) | 0.36 | 0.10 | **< .001** |
| Assessment time (time; knowledge vs. recall) | -23.54 | 1.42 | **< .001** |
| Knowledge type (Type; verbatim vs. gist) | 22.29 | 1.42 | **< .001** |
| Condition × representation | -2.08 | 4.14 | .616 |
| Condition × GL | 0.05 | 0.19 | .802 |
| Condition × time | -4.41 | 2.84 | .120 |
| Condition × type | -4.00 | 2.84 | .159 |
| Representation × GL | 0.80 | 0.19 | **< .001** |
| Representation × time | -1.27 | 2.84 | .655 |
| Representation × type | -1.84 | 2.84 | .518 |
| GL × time | 0.08 | 0.13 | .552 |
| GL × type | 0.10 | 0.13 | .445 |
| Time × type | 15.99 | 2.84 | **< .001** |
| Condition × representation × GL | -0.07 | 0.39 | .847 |
| Condition × representation × time | -0.25 | 5.67 | .964 |
| Condition × representation × type | 4.54 | 5.67 | .424 |
| Condition × GL × time | -0.17 | 0.26 | .531 |
| Condition × GL × type | -0.43 | 0.26 | .101 |
| Condition × time × type | 6.02 | 5.67 | .289 |
| Representation × GL × time | 0.45 | 0.26 | .086 |
| Representation × GL × type | 0.44 | 0.26 | .095 |
| Representation × time × type | -3.86 | 5.67 | .496 |
| GL × time × type | 0.46 | 0.26 | .081 |
| Condition × representation × GL × time | 0.10 | 0.53 | .851 |
| Condition × representation × GL × type | 0.03 | 0.53 | .950 |
| Condition × representation × time × type | 6.49 | 11.35 | .567 |
| Condition × GL × time × type | -0.02 | 0.53 | .968 |
| Representation × GL × time × type | 1.06 | 0.53 | **.044** |
| 5-way interaction | 0.45 | 1.06 | .669 |
| Numeracy | -0.12 | 0.06 | **.041** |

*Note.* Results in boldface indicate significant predictors.

*Figure S1.* Gist and verbatim knowledge scores for numbers versus graphs with separate lines for people with high and low graph literacy, separate panels for T1 (upper panels a and b) and T2 (lower panels c and d), and separate panels for conditions “no choice” (left panels a and c) and “choice” (right panels b and d), based on the medication-only scoring scheme described above. Error bars represent one standard error of the mean.


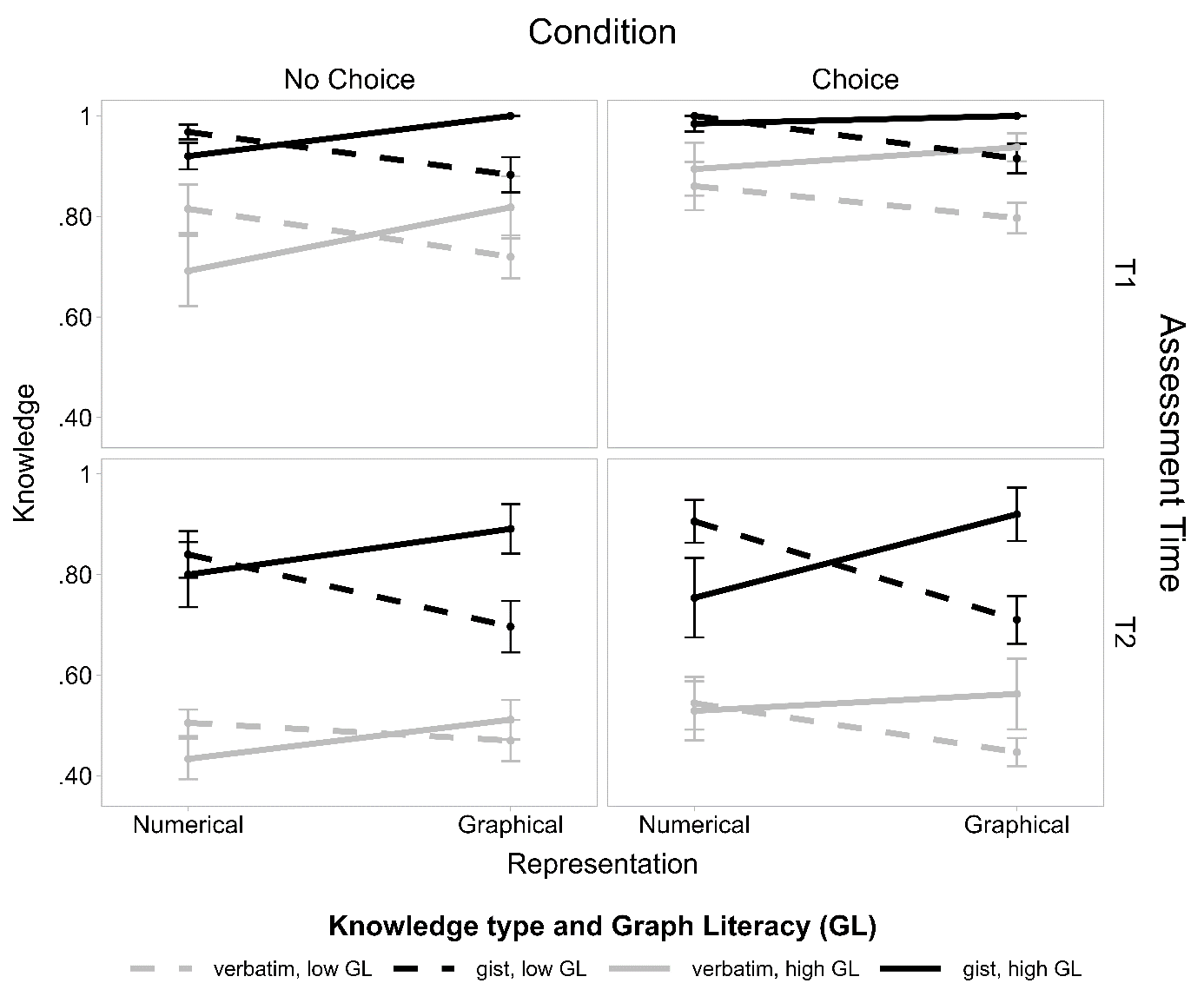


**d**

**c**

**b**

**a**

**Scoring of Missing Data**

In the analyses reported in the main text, we scored missing answers to knowledge, graph literacy, and numeracy items as incorrect. Overall, 80% of participants had no missing data at all on these questionnaires and further 13% had only two or less missing answers across the 48 items, indicating a low prevalence of missing answers. People who had at least one missing answer (*n* = 32) did not differ from those who had no missing answer (*n* = 128) with respect to age, gender, graph literacy, numeracy, and gist or verbatim knowledge (*p*s > .250). To test whether the scoring of missing answers affected the results, we reran all analyses, treating missing answers as missing data.

What is Chosen Predominantly, and Who Chose Graphs Rather Than Numbers?

Consistent with the results reported in the main text, the participants who chose the numerical representation did not differ from the participants who chose the graphical representation. This held true for graph literacy, *t*_Welch_(43.20) = 0.783, *p* = .438 and numeracy, *t*(76) = 0.078, *p* = .934. The analysis with regard to age and gender are the same as in the main text.

Does Choice Foster Comprehension?

The results of the mixed-effects model with knowledge as outcome variable is reported in Table S1. The results were similar to those reported in the main text.

Table S1. Results of the mixed-effects model when scoring missing answers as missing data

| Predictor | *b* | *SE* | *p* |
| --- | --- | --- | --- |
| Intercept | 72.76 | 1.07 | **< .001** |
| Condition (no choice vs. choice) | 2.83 | 2.15 | .189 |
| Representation (numerical vs. graphical) | -4.90 | 2.15 | **.023** |
| Graph Literacy (GL) | 0.38 | 0.10 | **< .001** |
| Assessment time (time; knowledge vs. recall) | -21.28 | 1.44 | **< .001** |
| Knowledge type (Type; verbatim vs. gist) | 13.86 | 1.44 | **< .001** |
| Condition × representation | -4.37 | 4.29 | .309 |
| Condition × GL | 0.10 | 0.20 | .619 |
| Condition × time | -3.45 | 2.88 | .231 |
| Condition × type | -7.83 | 2.88 | **.007** |
| Representation × GL | 0.78 | 0.20 | **< .001** |
| Representation × time | -2.68 | 2.88 | .352 |
| Representation × type | -3.19 | 2.88 | .268 |
| GL × time | -0.00 | 0.13 | .989 |
| GL × type | 0.03 | 0.13 | .809 |
| Time × type | 19.74 | 2.88 | **< .001** |
| Condition × representation × GL | 0.13 | 0.40 | .741 |
| Condition × representation × time | -2.82 | 5.75 | .625 |
| Condition × representation × type | 2.39 | 5.75 | .678 |
| Condition × GL × time | -0.08 | 0.27 | .778 |
| Condition × GL × type | -0.30 | 0.27 | .261 |
| Condition × time × type | 9.77 | 5.75 | .090 |
| Representation × GL × time | 0.31 | 0.27 | .251 |
| Representation × GL × type | 0.16 | 0.27 | .556 |
| Representation × time × type | -6.92 | 5.75 | .230 |
| GL × time × type | 0.35 | 0.27 | .196 |
| Condition × representation × GL × time | 0.08 | 0.54 | .881 |
| Condition × representation × GL × type | 0.63 | 0.54 | .245 |
| Condition × representation × time × type | 3.44 | 11.51 | .765 |
| Condition × GL × time × type | 0.29 | 0.54 | .590 |
| Representation × GL × time × type | 0.67 | 0.54 | .210 |
| 5-way interaction | 0.49 | 1.07 | .650 |
| Numeracy | -0.12 | 0.07 | .063 |

*Note.* Results in boldface indicate significant predictors.

Does Choice Increase Ratings of Accessibility and/or Attractiveness?

The results of the mixed-effects models with accessibility and attractiveness as outcome variables are reported in Tables S2 and S3. The results were similar to those reported in the main text.

Table S2. Results of the mixed-effects model with accessibility as outcome when scoring missing answers as missing data

| Predictor | *b* | *SE* | *p* |
| --- | --- | --- | --- |
| Intercept | 3.30 | 0.06 | **< .001** |
| Condition (no choice vs. choice) | 0.17 | 0.11 | .125 |
| Representation (numerical vs. graphical) | 0.08 | 0.11 | .463 |
| Graph Literacy (GL) | 0.00 | 0.01 | .700 |
| Numeracy (Num) | 0.00 | 0.00 | .841 |
| Condition × representation | 0.57 | 0.22 | **.012** |
| Condition × GL | 0.02 | 0.01 | .075 |
| representation × GL | 0.00 | 0.01 | .896 |
| 3-way interaction | 0.00 | 0.02 | .845 |

Table S3. Results of the mixed-effects model with attractiveness as outcome when scoring missing answers as missing data

| Predictor | *b* | *SE* | *p* |
| --- | --- | --- | --- |
| Intercept | 3.49 | 0.06 | **< .001** |
| Condition (no choice vs. choice) | 0.30 | 0.12 | **.014** |
| Representation (numerical vs. graphical) | 0.52 | 0.12 | **< .001** |
| Graph Literacy (GL) | 0.00 | 0.01 | .480 |
| Numeracy (Num) | 0.00 | 0.00 | .356 |
| Condition × representation | -0.19 | 0.24 | .433 |
| Condition × GL | 0.00 | 0.01 | .805 |
| representation × GL | -0.01 | 0.01 | .344 |
| 3-way interaction | 0.00 | 0.02 | .960 |

**Mixed-Effects Model with Numeracy as Predictor**

In our analysis on the effect of choice on comprehension reported in the main text, we included graph literacy as a predictor in the model and numeracy as a covariate. To test how robust our results are depending on the decision whether to include numeracy as a covariate vs. predictor, we conducted the same mixed-effects model reported in the main text, but included numeracy as a predictor (i.e., included the interactions of numeracy with all other predictors and interactions).

Does Choice Foster Comprehension?

The full results for knowledge as outcome are presented in Table S4. The results in this model are similar to the ones reported in the main text, except for the main effect of numeracy and the interaction effect of condition and knowledge type, which are not significant anymore. In addition, there was a significant positive three-way interaction of representation, time, and numeracy and a significant negative four-way interaction of condition, knowledge type, graph literacy, and numeracy.

Table S4. Results of the linear mixed-effects model with numeracy as a predictor

| Predictor | *b* | *SE* | *p* |
| --- | --- | --- | --- |
| Intercept | 71.67 | 1.16 | **< .001** |
| Condition (no choice vs. choice) | 2.26 | 2.32 | .329 |
| Representation (numerical vs. graphical) | -4.71 | 2.32 | **.043** |
| Graph Literacy (GL) | 0.34 | 0.11 | **.002** |
| Numeracy (Num) | -0.12 | 0.07 | .078 |
| Assessment time (time; knowledge vs. recall) | -21.33 | 1.48 | **< .001** |
| Knowledge type (Type; verbatim vs. gist) | 14.84 | 1.48 | **< .001** |
| Condition × representation | -4.85 | 4.63 | .296 |
| Condition × GL | 0.06 | 0.22 | .771 |
| representation × GL | 0.75 | 0.22 | **.001** |
| Condition × Num | 0.11 | 0.13 | .417 |
| representation × Num | 0.04 | 0.13 | .791 |
| GL × Num | 0.01 | 0.01 | .135 |
| Condition × time | -3.07 | 2.96 | .299 |
| representation × time | -4.10 | 2.96 | .166 |
| GL × time | 0.01 | 0.14 | .944 |
| Num × time | -0.06 | 0.09 | .498 |
| Condition × type | -4.42 | 2.96 | .136 |
| representation × type | -2.45 | 2.96 | .407 |
| GL × type | 0.09 | 0.14 | .536 |
| Num × type | -0.09 | 0.09 | .311 |
| time × type | 20.00 | 2.96 | **< .001** |
| Condition × representation × GL | 0.16 | 0.43 | .706 |
| Condition × representation × Num | 0.01 | 0.27 | .971 |
| Condition × GL × Num | 0.01 | 0.01 | .588 |
| representation × GL × Num | 0.00 | 0.01 | .802 |
| Condition × representation × time | -3.84 | 5.92 | .517 |
| Condition × GL × time | -0.15 | 0.28 | .595 |
| representation × GL × time | 0.08 | 0.28 | .778 |
| Condition × Num × time | 0.12 | 0.17 | .470 |
| representation × Num × time | 0.36 | 0.17 | **.038** |
| GL × Num × time | 0.00 | 0.01 | .775 |
| Condition × representation × type | -2.68 | 5.92 | .651 |
| Condition × GL × type | -0.33 | 0.28 | .225 |
| representation × GL × type | 0.26 | 0.28 | .349 |
| Condition × Num × type | -0.02 | 0.17 | .909 |
| representation × Num × type | 0.06 | 0.17 | .729 |
| GL × Num × type | -0.00 | 0.01 | .888 |
| Condition × time × type | 10.91 | 5.92 | .066 |
| representation × time × type | -6.26 | 5.92 | .291 |
| GL × time × type | 0.16 | 0.28 | .552 |
| Num × time × type | 0.04 | 0.17 | .815 |
| Condition × representation × GL × Num | 0.01 | 0.03 | .822 |
| Condition × representation × GL × time | -0.02 | 0.55 | .964 |
| Condition × representation × Num × time | 0.23 | 0.34 | .506 |
| Condition × GL × Num × time | -0.01 | 0.02 | .632 |
| representation × GL × Num × time | 0.03 | 0.02 | .107 |
| Condition × representation × GL × type | 0.38 | 0.55 | .491 |
| Condition × representation × Num × type | -0.23 | 0.34 | .494 |
| Condition × GL × Num × type | -0.09 | 0.02 | **< .001** |
| representation × GL × Num × type | 0.03 | 0.02 | .124 |
| Condition × representation × time × type | -2.91 | 11.83 | .806 |
| Condition × GL × time × type | 0.35 | 0.55 | .527 |
| representation × GL × time × type | 0.81 | 0.55 | .142 |
| Condition × Num × time × type | -0.17 | 0.34 | .619 |
| representation × Num × time × type | -0.21 | 0.34 | .535 |
| GL × Num × time × type | -0.01 | 0.02 | .747 |
| Condition × representation × GL × Num × time | 0.02 | 0.04 | .520 |
| Condition × representation × GL × Num × type | -0.02 | 0.04 | .534 |
| Condition × representation × GL × time × type | -0.21 | 1.10 | .850 |
| Condition × representation × Num × time × type | 0.44 | 0.68 | .517 |
| Condition × GL × Num × time × type | -0.04 | 0.04 | .287 |
| representation × GL × Num × time × type | -0.03 | 0.04 | .454 |
| 6-way interaction | 0.08 | 0.07 | .286 |

Does Choice Increase Ratings of Accessibility and/or Attractiveness?

The results of the mixed-effects models with accessibility and attractiveness as outcome variables are reported in Tables S5 and S6. The results are similar to those reported in the main text.

Table S5. Results of the mixed-effects model with accessibility as outcome and with numeracy as a predictor

| Predictor | *b* | *SE* | *p* |
| --- | --- | --- | --- |
| Intercept | 3.31 | 0.06 | **< .001** |
| Condition (no choice vs. choice) | 0.19 | 0.12 | .112 |
| Representation (numerical vs. graphical) | 0.05 | 0.12 | .688 |
| Graph Literacy (GL) | -0.00 | 0.01 | .868 |
| Numeracy (Num) | -0.00 | 0.00 | .971 |
| Condition × representation | 0.53 | 0.24 | **.028** |
| Condition × GL | 0.02 | 0.01 | .096 |
| representation × GL | 0.00 | 0.01 | .973 |
| Condition × Num | -0.00 | 0.01 | .695 |
| representation × Num | 0.00 | 0.01 | .624 |
| GL × Num | -0.00 | 0.00 | .551 |
| Condition × representation × GL | 0.01 | 0.02 | .656 |
| Condition × representation × Num | -0.01 | 0.01 | .319 |
| Condition × GL × Num | -0.00 | 0.00 | .873 |
| representation × GL × Num | 0.00 | 0.00 | .628 |
| 4-way interaction | 0.00 | 0.00 | .496 |

Table S6. Results of the mixed-effects model with attractiveness as outcome and with numeracy as a predictor

| Predictor | *b* | *SE* | *p* |
| --- | --- | --- | --- |
| Intercept | 3.49 | 0.06 | **< .001** |
| Condition (no choice vs. choice) | 0.26 | 0.13 | **.049** |
| Representation (numerical vs. graphical) | 0.52 | 0.13 | **< .001** |
| Graph Literacy (GL) | -0.00 | 0.01 | .605 |
| Numeracy (Num) | -0.00 | 0.00 | .408 |
| Condition × representation | -0.09 | 0.26 | .723 |
| Condition × GL | 0.00 | 0.01 | .949 |
| representation × GL | -0.01 | 0.01 | .359 |
| Condition × Num | 0.00 | 0.01 | .788 |
| representation × Num | -0.00 | 0.01 | .876 |
| GL × Num | 0.00 | 0.00 | .689 |
| Condition × representation × GL | 0.01 | 0.02 | .749 |
| Condition × representation × Num | -0.02 | 0.02 | .207 |
| Condition × GL × Num | 0.00 | 0.00 | .318 |
| representation × GL × Num | -0.00 | 0.00 | .726 |
| 4-way interaction | -0.00 | 0.00 | .295 |

**Distribution of Graph Literacy and Numeracy**

In our study, graph literacy, but not numeracy, was positively associated with knowledge. One possible reason could be that the distribution of numeracy was very skewed in our sample. To make this issue transparent, Figure S2 therefore presents the distributions of graph literacy and numeracy in our sample.

*
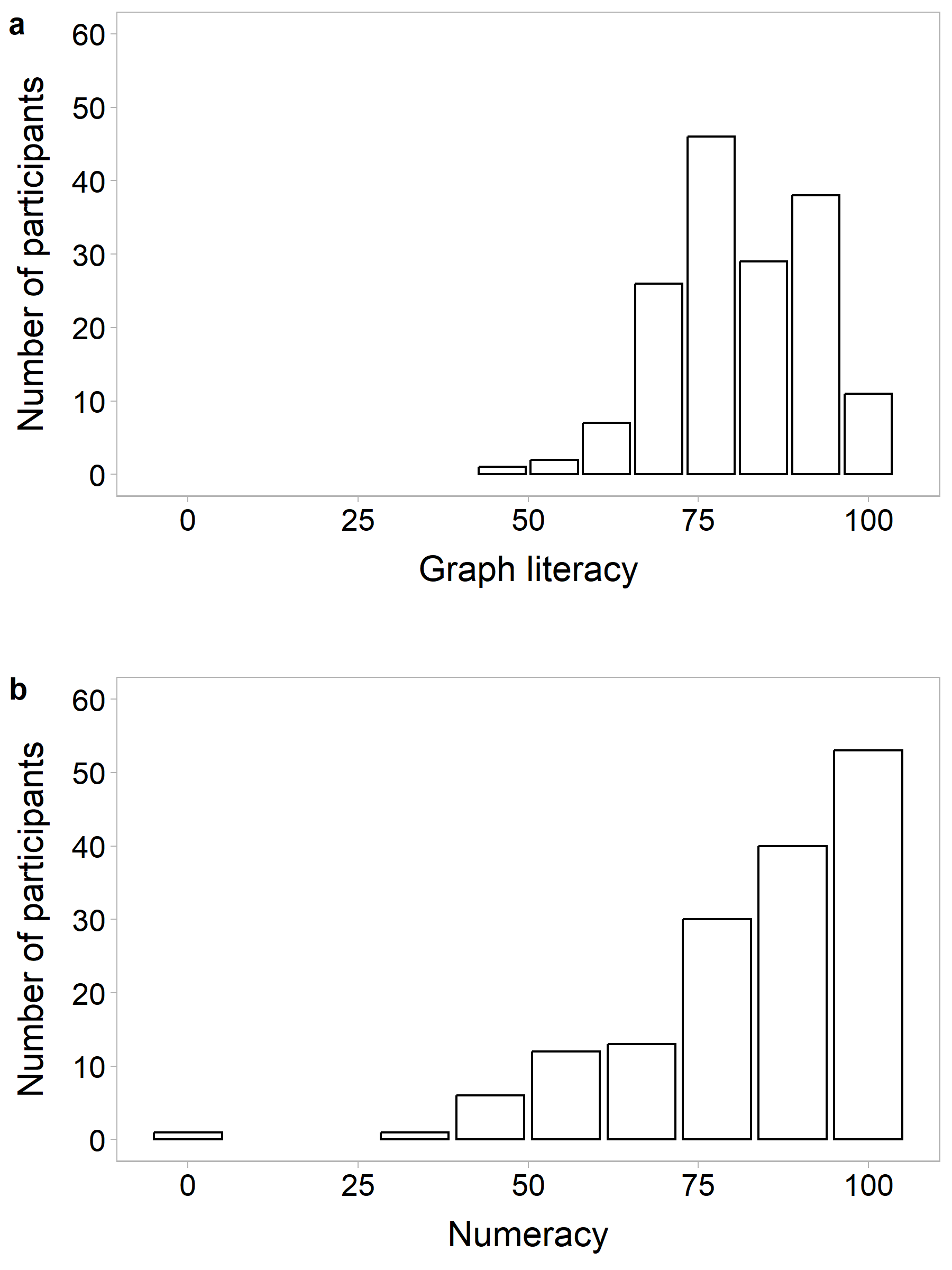
Figure S2*. Distribution of (a) graph literacy and (b) numeracy in our sample.

**Materials Used in the Study**

**Title Page of “Choice” Condition**

After reading the title page, participants are provided with medical information in either the graphical or the numerical format, depending on their choice. Subsequently, there are asked to complete in the questionnaire.

ID: _________

Date:________________

Age: ________________ years

Sex: f | w

Field of study: ________________

Dear participant!

This is a study in collaboration between the University of Granada and the Max Planck Institute for Human Development in Berlin, Germany. We are interested in your judgments regarding a variety of painkillers. To this end, we will provide you with information regarding these painkillers and ask you a variety of questions about them.

Please note that all of this is a summary of actual, large clinical trials that compared the benefits and side effects of the painkillers to a placebo (“sugar pill”) condition.

Please decide how you like to receive the information and tell the experimenter, so that she can provide you with it.

| **numerically**  *it will look approximately like this:* | | OR | **graphically**  *it will look approximately like this:* |
| --- | --- | --- | --- |
| Benefits:  Side effects: |  |  |  |

*Now please turn the page to receive the questions.*

**Title Page of “No-Choice” Condition**

After reading the title page, participants are provided with medical information in either the graphical or the numerical format, depending on the random allocation. Subsequently, there are asked to complete in the questionnaire.

ID: _________

Date:________________

Age: ________________ years

Sex: f | w

Field of study: ________________

Dear participant!

This is a study in collaboration between the University of Granada and the Max Planck Institute for Human Development in Berlin, Germany. We are interested in your judgments regarding a variety of painkillers. To this end, we will provide you with information regarding these painkillers and ask you a variety of questions about them.

Please note that all of this is a summary of actual, large clinical trials that compared the benefits and side effects of the painkillers to a placebo (“sugar pill”) condition.

The experimenter will now provide you with the information.

*Now please turn the page to receive the questions.*

**Medical Information (Numbers Condition)**

**Medical Information (Graphs Condition)**

**Questionnaire (identical for both conditions)**

| For which drug was a pain reduction of more than 50% most frequently observed? |
| --- |
| - Aspirin - Paracetamol - Ibuprofen |
| Which drug caused side effects most frequently? |
| - Aspirin - Paracetamol - Ibuprofen |
| Which drug caused side effects least frequently? |
| - Aspirin - Paracetamol - Ibuprofen |

| Which painkiller was best **overall**? |
| --- |
| - Aspirin - Paracetamol - Ibuprofen |
| Which painkiller was worst **overall**? |
| - Aspirin - Paracetamol - Ibuprofen |

If 10 people take **Aspirin**, how many of them will experience a **pain reduction** of more than 50%?

**________________**

If 10 people take **Ibuprofen**, how many of them will experience **side effects**?

**________________**

If 10 people take **the placebo that was compared to Paracetamol**, how many of them will experience a **pain reduction** of more than 50%?

**________________**

If 10 people **take the placebo that was compared to Aspirin**, how many of them will experience **side effects**?

**________________**

If 10 people take **Ibuprofen**, how many of them will experience a **pain reduction** of more than 50%, **which they would not have experienced by the respective placebo**?

**________________**

If 10 people take **Aspirin**, how many of them will experience a **pain reduction** of more than 50%, **which they would not have experienced by the respective placebo**?

**________________**

If 10 people take **Aspirin**, how many of them will experience **side effects, which they would not have experienced by the respective placebo**?

**________________**

If 10 people take **Paracetamol**, how many of them will experience **side effects, which they would not have experienced by the respective placebo**?

**________________**

| How comprehensible was the information? | | | | | | |
| --- | --- | --- | --- | --- | --- | --- |
| not at all comprehensible | 🞏 | 🞏 | 🞏 | 🞏 | 🞏 | very comprehensible |

| How useful was the presented information? | | | | | | |
| --- | --- | --- | --- | --- | --- | --- |
| not at all useful | 🞏 | 🞏 | 🞏 | 🞏 | 🞏 | very useful |

| As how serious/respectable did you perceive presented information? | | | | | | |
| --- | --- | --- | --- | --- | --- | --- |
| not at all serious | 🞏 | 🞏 | 🞏 | 🞏 | 🞏 | very serious |

| Do you think that the information was intuitively accessible? | | | | | | |
| --- | --- | --- | --- | --- | --- | --- |
| not at all accessible | 🞏 | 🞏 | 🞏 | 🞏 | 🞏 | very accessible |

| How difficult was it for you to answer the questions with the represented information? | | | | | | |
| --- | --- | --- | --- | --- | --- | --- |
| not at all difficult | 🞏 | 🞏 | 🞏 | 🞏 | 🞏 | very difficult |

| How attractive was the representation of the information in your view? | | | | | |
| --- | --- | --- | --- | --- | --- |
|  | Not at all attractive |  |  |  | Very attractive |
| Overall impression | 🞏 | 🞏 | 🞏 | 🞏 | 🞏 |
| Colors | 🞏 | 🞏 | 🞏 | 🞏 | 🞏 |
| Imagery | 🞏 | 🞏 | 🞏 | 🞏 | 🞏 |
| Technical implementation | 🞏 | 🞏 | 🞏 | 🞏 | 🞏 |
| Size | 🞏 | 🞏 | 🞏 | 🞏 | 🞏 |
| Font size | 🞏 | 🞏 | 🞏 | 🞏 | 🞏 |
| Font | 🞏 | 🞏 | 🞏 | 🞏 | 🞏 |
| Composition | 🞏 | 🞏 | 🞏 | 🞏 | 🞏 |

*Now, please give the information the experimenter handed to you back to her.*

*There are a few more other questions that we’d like you to answer.*

Here is some information about cancer therapies.


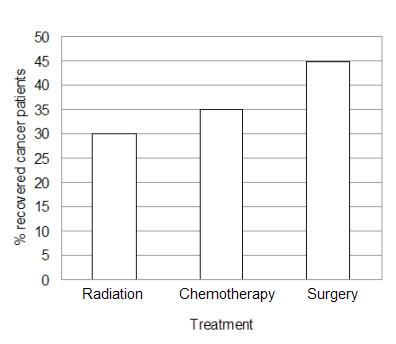


Q1. What percentage of patients recovered after chemotherapy?

%

Q2. What is the difference between the percentage of patients who recovered after a surgery and the percentage of patients who recovered after radiation therapy?

%

Here is some information about different forms of cancer.


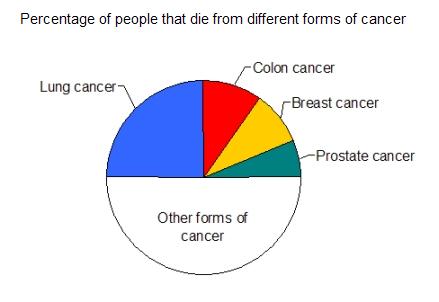


Q3. Of all the people who die from cancer, approximately what percentage dies from lung cancer?

%

Q4. Approximately what percentage of people who die from cancer die from colon cancer, breast cancer, and prostate cancer taken together?

%

Here is some information about an imaginary disease called Adeolitis.

Percentage of people with Adeolitis

**
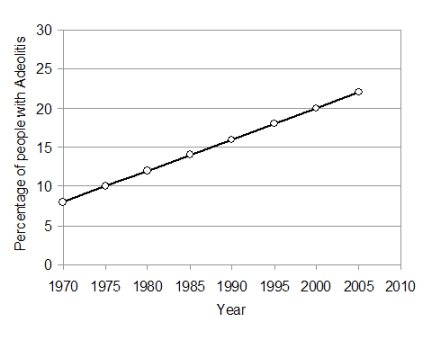
**

Q5. Approximately what percentage of people had Adeolitis in the year 2000?

%

Q6. When was the increase in the percentage of people with Adeolitis higher?

From 1975 to 1980………………………………………..1

From 2000 to 2005………………………………………..2

Increase was the same in both intervals…………………...3

Don’t know………………………………………………..4

Q7. According to your best guess, what will the percentage of people with Adeolitis be in the year 2010?

%

The following figure shows the number of men and women among patients with disease X. The total number of circles is 100.

**
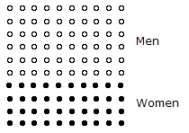
**

Q8. Of 100 patients with disease X, how many are women?

Q9. How many more men than women are there among 100 patients with disease X?

men

Q10. In a magazine you see two advertisements, one on page 5 and another on page 12. Each is for a different drug for treating heart disease, and each includes a graph showing the effectiveness of the drug compared to a placebo (sugar pill).


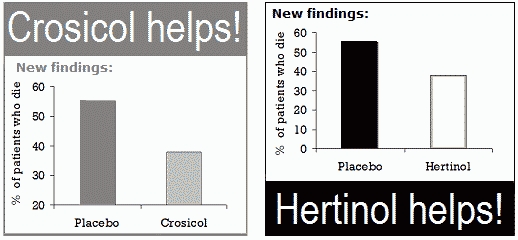


Compared to the placebo, which treatment leads to a larger decrease in the percentage of patients who die?

Crosicol………………………..1

Hertinol………………………..2

They are equal…………………3

Can’t say………………………4

Q11. In the newspaper you see two advertisements, one on page 15 and another on page 17. Each is for a different treatment of psoriasis, and each includes a graph showing the effectiveness of the treatment over time.

**
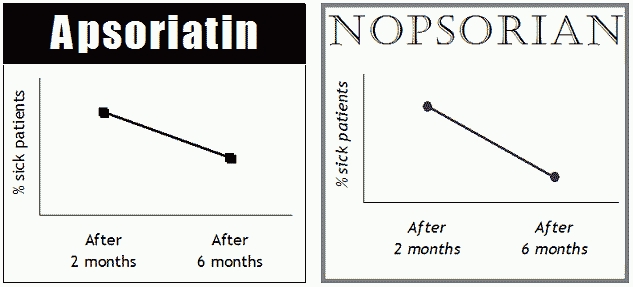
**

Which of the treatments contributes to a larger decrease in the percentage of sick patients?

Apsoriatin………………….1

Nopsorian………………….2

They are equal……………..3

Can’t say…………………...4

Q12. Here is some information about the imaginary diseases Coliosis and Tiosis.

**
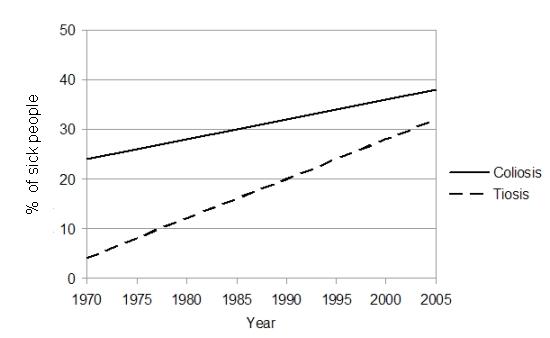
**

Between 1980 and 1990, which disease had a higher increase in the percentage of people affected?

Coliosis……………………………1

Tiosis……………………………...2

The increase was equal……………3

Can’t say…………………………..4

Q13. Here is some information about cancer therapies.


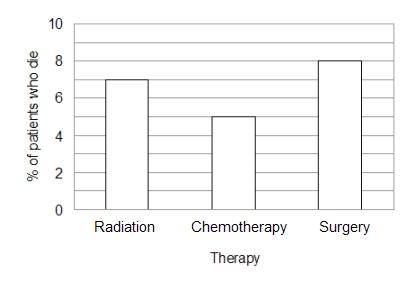


What is the percentage of cancer patients who die after chemotherapy?

%

Q14. Imagine that we flip a fair coin 1,000 times. What is your best guess about how many times the coin would come up heads in 1,000 flips?

_______ times out of 1,000

Q15. In the Bingo Lottery, the chance of winning a $10 prize is 1%. What is your best guess about how many people would win a $10 prize if 1,000 people each buy a single ticket to Bingo Lottery?

_______ person(s) out of 1,000

Q16. In Daily Times Sweepstakes, the chance of winning a car is 1 in 1,000. What percent of tickets to Daily Times Sweepstakes win a car?

_______ % of tickets

Q17. Imagine that we roll a fair, six-sided die 1,000 times. Out of 1,000 rolls, how many times do you think the die would come up even (2, 4, or 6)?

_______ times out of 1,000

Q18. Which of the following numbers represents the biggest risk of getting a disease?

a.- 1 in 10

b.- 1 in 100

c.- 1 in 1,000

Q19. Which of the following represents the biggest risk of getting a disease?

a.- 1%

b.- 5%

c.- 10%

Q20. If the chance of getting a disease is 10%, how many people would be expected to get the disease out of 1,000?

_______ people

Q21. If the chance of getting a disease is 20 out of 100, this would be the same as having a _______% chance of getting the disease.

Q22. If Person A’s chance of getting a disease is 1 in 100 in ten years, and Person B’s risk is double that of A, what is B’s risk?

_______
